# Development of *Bacillus methanolicus* methanol dehydrogenase with improved formaldehyde reduction activity

**DOI:** 10.1038/s41598-018-31001-8

**Published:** 2018-08-20

**Authors:** Jiyeun Yi, Jinhyuk Lee, Bong Hyun Sung, Du-Kyeong Kang, GyuTae Lim, Jung-Hoon Bae, Seung-Goo Lee, Sun Chang Kim, Jung-Hoon Sohn

**Affiliations:** 1 Department of Biological Sciences, Korea Advanced Institute of Science and Technology, Daejeon, 34141 South Korea; 2 Cell Factory Research Center, Korea Research Institute of Bioscience and Biotechnology, Daejeon, 34141 South Korea; 3 Genome Editing Research Center, Korea Research Institute of Bioscience and Biotechnology, Daejeon, 34141 South Korea; 4 School of Biotechnology, Korea University of Science and Technology, Daejeon, 34113 South Korea; 5 Synthetic Biology and Bioengineering Research Center, Korea Research Institute of Bioscience and Biotechnology, Daejeon, 34141 South Korea

## Abstract

Methanol dehydrogenase (MDH), an NAD^+^-dependent oxidoreductase, reversibly converts formaldehyde to methanol. This activity is a key step for both toxic formaldehyde elimination and methanol production in bacterial methylotrophy. We mutated decameric *Bacillus methanolicus* MDH by directed evolution and screened mutants for increased formaldehyde reduction activity in *Escherichia coli*. The mutant with the highest formaldehyde reduction activity had three amino acid substitutions: F213V, F289L, and F356S. To identify the individual contributions of these residues to the increased reduction activity, the activities of mutant variants were evaluated. F213V/F289L and F213V/F289L/F356S showed 25.3- and 52.8-fold higher catalytic efficiency (*k*_cat_/*K*_m_) than wild type MDH, respectively. In addition, they converted 5.9- and 6.4-fold more formaldehyde to methanol *in vitro* than the wild type enzyme. Computational modelling revealed that the three substituted residues were located at MDH oligomerization interfaces, and may influence oligomerization stability: F213V aids in dimer formation, and F289L and F356S in decamer formation. The substitutions may stabilise oligomerization, thereby increasing the formaldehyde reduction activity of MDH.

## Introduction

Increased atmospheric CO_2_ is a major cause of global warming and climate change. Many *in vitro* studies have examined single or multiple enzyme systems to reduce CO_2_ and convert it to valuable chemicals and fuels^1–3^. Converting CO_2_ into methanol using a multi-enzyme system is one of the most promising possible routes, recycling the greenhouse gas to efficiently produce a fuel alternative. In comparison to gas-based fuels (CO, methane, etc.) produced by the single-enzyme route, the liquid methanol produced by multi-enzyme reaction has a much higher energy capacity and is easier to transport^4^. Converting CO_2_ to methanol involves the consecutive reactions of three enzymes: formate dehydrogenase reduces CO_2_ to formate, formaldehyde dehydrogenase reduces formate to formaldehyde, and alcohol dehydrogenase (ADH) reduces formaldehyde to methanol. This sequence has been studied in various reaction conditions: with encapsulation of the three enzymes in a porous silica sol-gel matrix^5^, immobilization of the enzymes and their cofactor β-nicotinamide adenine dinucleotide (NAD^+^) in polystyrene particles and TiO_2_ nanoparticles^6,7^, with artificial photosynthesis, by combining a photochemical cofactor reducing system with the enzymatic conversion system^8^, and in a thermodynamic feasibility investigation of multiple enzyme reactions^9^.

Methanol dehydrogenase (MDH) is an oxidoreductase that catalyses the reversible interconversion of formaldehyde and methanol^10^. Two classes of MDHs are expressed by methylotrophic bacteria: the pyrroloquinoline quinone-containing MDHs, which are found mostly in Gram-negative and strictly aerobic methylotrophs, and the NAD(P) + -dependent cytoplasmic MDHs, commonly found in thermophilic, Gram-positive bacteria^11^. Although the former has been previously used to convert CO_2_ to methanol^12^, the latter is more suitable because of their use of reduced β-nicotinamide adenine dinucleotide (NADH) as a cofactor^13^.

The NAD-dependent MDHs belong to ADH group III^14^. Three NAD-dependent MDHs are found in *Bacillus methanolicus* MGA3 and PB1^15^. MDH2 and MDH3 on the chromosome of MGA3 are 96% homologous to each other and approximately 60% to MDH on the plasmid^16^. MDH from *B. methanolicus* C1, mostly studied for structures and characteristics, contains one zinc ion and two magnesium ions at its active site^17,18^ and acts as a decamer^19^. Methanol oxidation by MDH is important for methylotrophic growth. However, kinetic substrate affinity and V_max_ values of MDH is higher for formaldehyde than for methanol^16,20^. Although the biological significance of formaldehyde reduction by MDH is not clear, MDH can be used for the reduction of formaldehyde in a sequential reaction for the conversion of carbon dioxide to methanol.

Most studies on CO_2_ conversion have focused on finding the optimal reaction conditions for commercial enzymes. Generally, the methanol yields reported under regular conditions by naturally evolved enzymes have been very low^9^, indicating the need to develop more efficient CO_2_ conversion enzymes. However, since the MDH protein structure at the atomic level has not been determined, it is difficult to develop novel enzymes using a rational design system.

In this study, we generated mutants of MDH of *B. methanolicus* C1 by directed evolution and screened them for improved formaldehyde reduction activity. The influences of the mutant residues on the increased reduction activity were simulated by computational structure modelling.

## Results and Discussion

### Screening of MDH mutants with increased formaldehyde reduction activity

**Figure 1:**
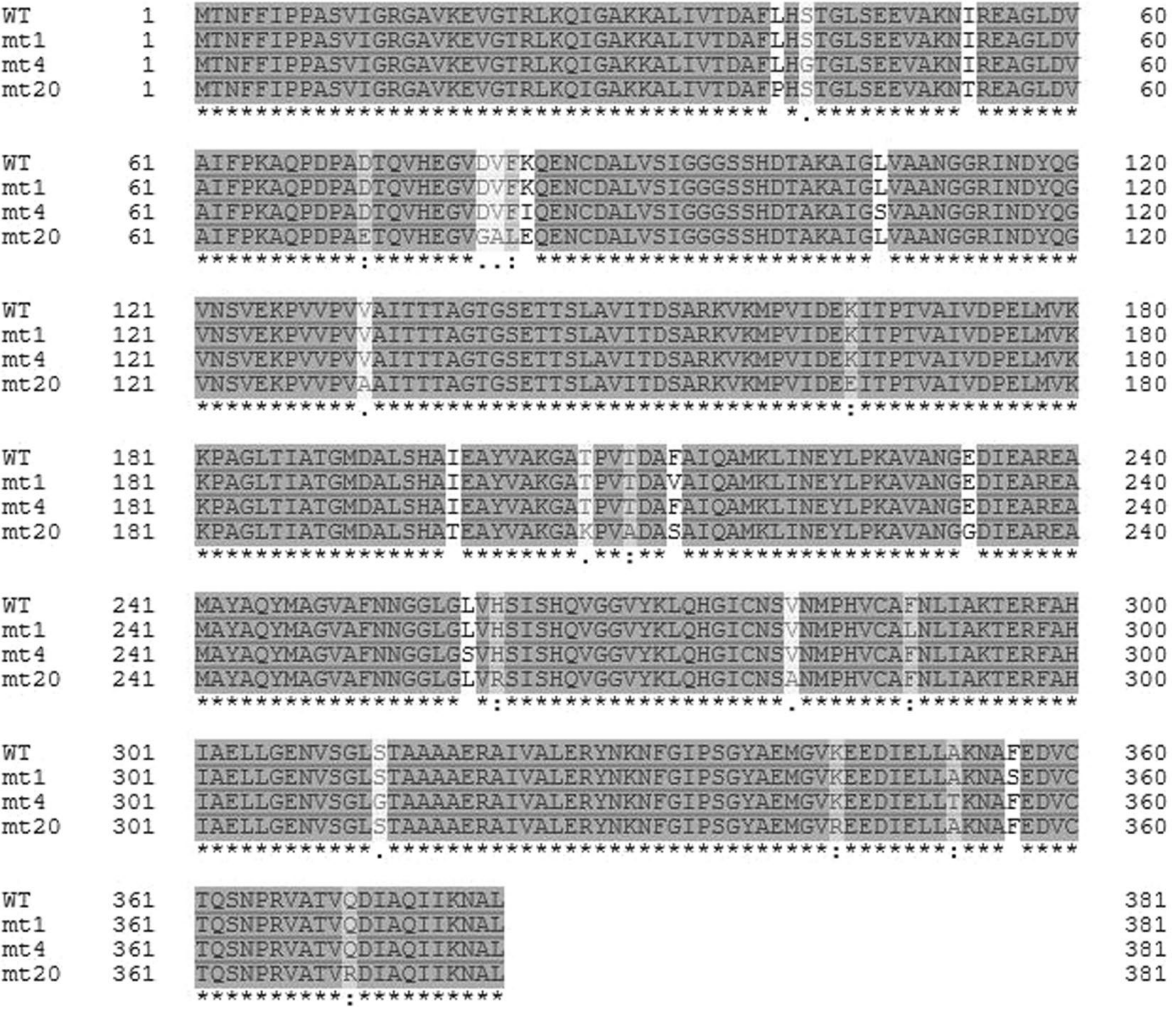
Amino acid sequences of the screened MDH mutants. MDHmt1, MDHmt4, and MDHmt20 had three, six, and 18 substituted amino acids, respectively. Dots indicate residues identical to the wild type (WT) enzyme.

To develop a screening system for MDH mutants with increased formaldehyde reduction activity, we exploited the cytotoxicity differences between formaldehyde and methanol in *Escherichia coli*. The minimal inhibitory concentration (MIC) of formaldehyde in *E. coli* is approximately 5 mM^21^, while that of methanol is >3 M^22^. When we treated *E. coli* BL21 (DE3) cells expressing wild type MDH with various concentration of formaldehyde, cell growth was arrested at concentrations >2 mM (data not shown). Therefore, we expected that if mutant MDH variants efficiently converted formaldehyde to methanol, the host cells would be able to survive in a medium containing 3 mM formaldehyde and would dominate the mutant library population. To identify MDH mutants with higher reduction activity, error-prone polymerase chain reaction (PCR) was performed using *mdh* from *B. methanolicus* C1, with 2–8 bp substitutions per kb. The PCR products were cloned into the pET21b plasmid under the control of a T7 promoter, and approximately 3 × 10^4^ BL21 (DE3) transformants were grown in media containing 3 mM formaldehyde. After 24 h of culture, cells were streaked on agar plates and plasmids in single colonies were isolated and sequenced to identify mutations in *mdh*. We selected 20 colonies for analysis, and 18 harboured the same three mutations (F213V, F289L, and F356S). This mutant was designated MDHmt1. The two other identified mutants had six and 18 amino acid substitutions, and were named MDHmt4 and MDHmt20, respectively. Their amino acid sequence changes are shown in Fig. 1.

### Tolerance to formaldehyde by mutant MDHs

**Figure 2:**
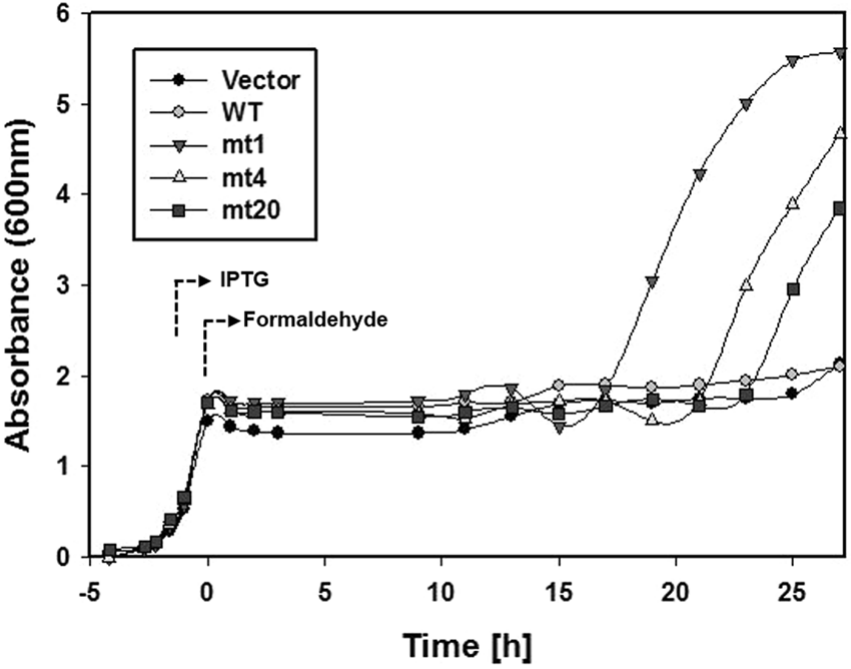
Growth rates of *E. coli* cells expressing mutant MDH in 3 mM formaldehyde. Isopropyl β-D-1-thiogalactopyranoside (IPTG) was added at an OD_600_ of 0.6 to induce MDH expression, and after 1 h, 3 mM formaldehyde was added. Vector, negative control with no *mdh* gene; WT, wild type *mdh* gene; mt1, MDHmt1 gene; mt4, MDHmt4 gene; mt20, MDHmt20 gene.

To analyse their tolerance to formaldehyde, *E. coli* BL21 (DE3) cells expressing MDHmt1, MDHmt4, and MDHmt20 were cultivated in 2YT media containing 3 mM formaldehyde, respectively. The cells displayed growth arrest until 15 h after formaldehyde addition; however, cells expressing MDHmt1, MDHmt4, and MDHmt20 started to grow after 17, 21, and 23 h, respectively (Fig. 2). The delayed growth of the cells may be due to formaldehyde evaporation and concentration decrease by MDH mutants. However, wild type MDH cells did not grow until after 27 h, at which time MDHmt1 had already reached the stationary phase. This observation of the cell growth at 3 mM formaldehyde concentration was due to formaldehyde reduction by MDH mutants. To confirm that the formaldehyde tolerance was indeed caused by the mutations, the experiments were repeated independently in triplicate, and the same observations were made. We concluded that the MDH mutants reduced formaldehyde to methanol faster than the wild type. Therefore, host cells expressing MDH mutants escaped from the growth arrest faster than those expressing the wild type.

### Site-directed mutagenesis of MDH

MDHmt1 contained three amino acid substitutions: F213V, F289L, and F356S. Interestingly, all substitutions changed phenylalanine residues, which contain bulky benzyl side groups, to smaller residues. Primary sequence alignment of the MDH protein from *B. methanolicus* C1 revealed that it is 97% identical to MDH of MGA3^16^. When three substituted sites were compared with those of MDHs of *B. methanolicus* MGA3 strain, it was observed that Phe^289^ and Phe^213^ were conserved in MDH. However, the Phe^356^ residue was changed to tyrosine in MDH of MGA3 and methionine in MDH2 and MDH3^16^. To analyse which amino acid changes contributed to increased formaldehyde reduction activity, the three mutations were introduced separately and in combination to wild type MDH to form F213V, F289L, F356S, F213V/F289L, F289L/F356S, F213V/F356S, and F213V/F289L/F356S variants.

To examine the kinetic properties of formaldehyde reduction for the wild type and mutant forms of MDH, we determined the Michaelis-Menten kinetic parameters (Table 1) *in vitro* using the recombinantly produced and purified enzymes. The kinetic parameters (K_M_ and Vmax) of wild type MDH from strain C1 was similar to that of MDH from MGA3^16^. The F213V mutant showed a 3.4-fold higher catalytic efficiency (*k*_cat_/*K*_M_) than wild type MDH. All mutants with two amino acid substitutions exhibited higher catalytic efficiencies than wild type MDH. The mutant containing all three amino acid substitutions exhibited the highest catalytic efficiency, with a 52.8-fold increase compared with wild type MDH.

**Table 1:** Kinetic parameters of MDH mutant variants.

	*K*_M_^a^ [mM]	*k*_cat_ [hr^−1^]	*k*_cat_/*K*_M_ [hr^−1^mM^−1^]	Relative efficiency^b^
WT	1.37 ± 0.63	208.3 ± 28.5	165.9 ± 44.0	1
F213V	2.77 ± 0.48	1564.4 ± 90.9	572.0 ± 62.2	3.4
F289L	2.98 ± 0.28	226.4 ± 4.2	76.3 ± 5.8	0.5
F356S	0.75 ± 0.17	50.0 ± 7.0	68.0 ± 6.4	0.4
F213V/F289L	0.58 ± 0.01	2452.8 ± 1.4	4200.4 ± 46.8	25.3
F289L/F356S	0.17 ± 0	150.8 ± 0.3	893.1 ± 19.6	5.4
F213V/F356S	1.28 ± 0.12	2091.2 ± 25.9	1637.7 ± 131.0	9.9
F213V/F289L/F356S	0.26 ± 0	2292.1 ± 8.0	8767.7 ± 21.4	52.8

^a^Formaldehyde as a substrate. ^b^Relative efficiency = [*k*_cat_/*K*_M_ (mutant)]/[*k*_cat_/*K*_M_ (wild type)].

The data shown are the means and standard deviations of three independent experiments.

**Figure 3:**
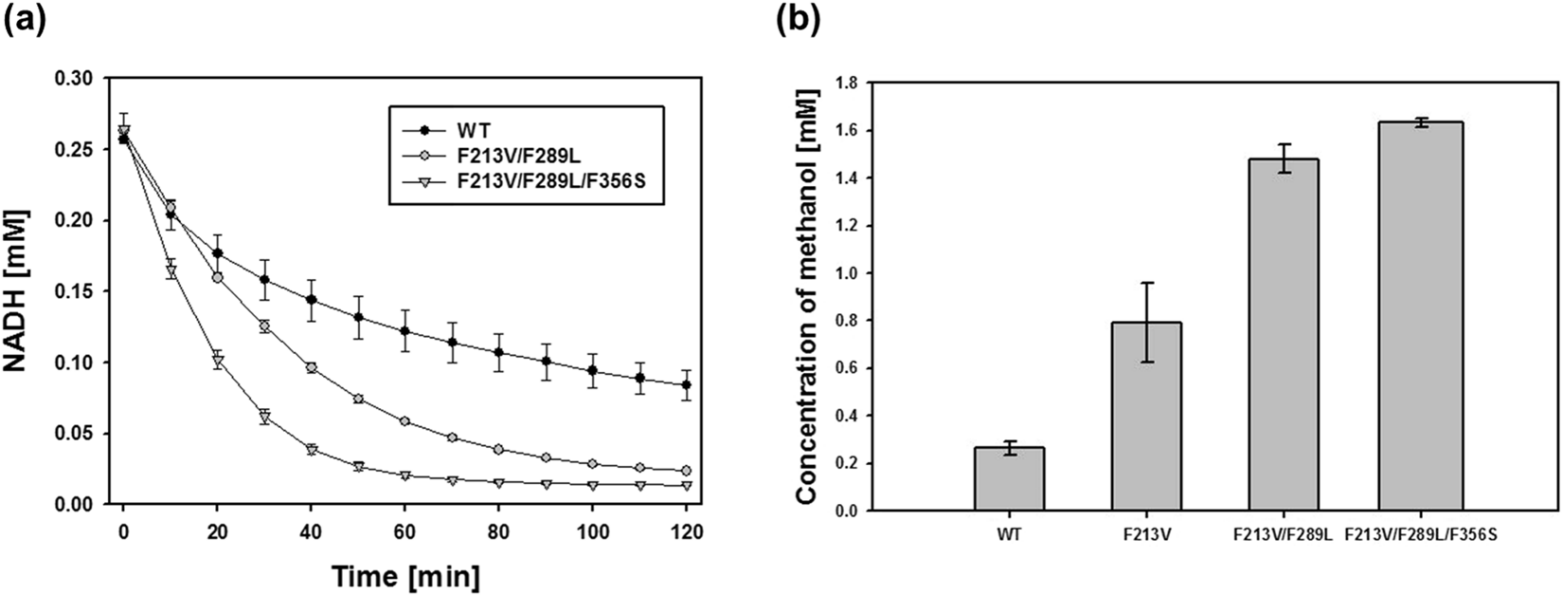
Formaldehyde reduction activity of MDH variants. (**a**) Concentration of the cofactor NADH during the reduction reaction. WT, wild type MDH. (**b**) After reduction by each MDH variant for 1 h at 50 °C, methanol levels were measured by HPLC. The data represent the mean value of three independent experiments.

Next, we measured the NADH reduction rate for F213V/F289L and F213V/F289L/F356S (Fig. 3a), which showed the highest catalytic efficiency for formaldehyde reduction. The time required for NADH to decrease by half in the presence of the mutant MDHs (100 μg) was shorter than that for the wild type enzyme, taking 15, 29, and 53 min for the F213V/F289L/F356S mutant, the F213V/F289L mutant, and wild type MDH, respectively. When we measured the conversion ratio of formaldehyde to methanol for wild type MDH and the two mutants by high-performance liquid chromatography (HPLC), 0.26 ± 0.03, 0.79 ± 0.16, 1.48 ± 0.06, and 1.63 ± 0.02 mM of methanol was produced by wild type MDH, F213V, F213V/F289L, and F213V/F289L/F356S, respectively, from 30 mM formaldehyde in a 4 h reaction at room temperature (Fig. 3b). Also, to confirm the correlation between the concentration change of formaldehyde and methanol, the consumption of formaldehyde and the production of methanol by wile type MDH and F213V/F289L/F356S was measured in a time-dependent manner (Fig. S1). The conversion rates of formaldehyde under the tested conditions were low compared to the kinetic parameters, and the reason for this is unclear. However, one possible reason is that the reduction reaction in Fig. 3b was performed at room temperature to prevent formaldehyde and methanol evaporation, although the optimal temperature for *B. methanolicus* MDH activity is 50 °C^23^ and kinetic parameters were determined at this temperature. Increase in formaldehyde reduction further could be achieved by an additional screening of mutant MDHs with increased formaldehyde reduction activity, continuous supply of the substrate, and removal of products to prevent reversible methanol oxidation^11^.

### Homology modelling of MDH

To examine the roles of the amino acid residues changed in MDH mutants, protein structure analysis was performed. However, because the experimental structure for MDH has not been characterized, we built a monomeric structure of MDH using Pseudo Quadratic Restraints with Simulated Annealing (PQR-SA) homology modelling (Fig. S2a). The generated structure was validated using various protein quality scores, including the radius of gyration (Fig. S2b) and a score radar chart (Fig. S2c). *B. methanolicus* MDH shares its highest sequence identity (0.43) with *Zymomonas mobilis* MDH (Protein Data Bank (PDB): 3OX4.A). Maximum potential sequence identity with the template combination used was 0.77. The radius of gyration was slightly smaller than that of similarly sized proteins (Fig. S2b), indicating a more compact protein structure. The MDH homology model had pack1, clash, WhatRama, MolRama, ProRama, and rotamer protein quality scores within 50% (Fig. S2c). Therefore, the generated homology model was valid for use in further structural analyses.

Since the developed homology model contains no information on interacting ligands, we performed binding site prediction for the cofactor NAD^+^ using the Dockable Pocket Site Prediction (DPSP) method (Fig. S3). In total, 40 residues were identified as potential NAD^+^ interacting residues (residue numbers 37, 39, 40, 43, 45, 68, 69, 70, 94, 95, 96, 97, 98, 100, 136, 137, 140, 142, 145, 147, 148, 149, 158, 161, 177, 180, 181, 182, 185, 188, 189, 192, 196, 252, 257, 261, 265, 274, 275, and 276). Two analogue ligands, nicotinamide-adenine-dinucleotide phosphate and 5,6-dihydroxy-NADP, shared interacting residues in the binding pocket with NAD^+^. The three mutation sites had no direct connection to the NAD^+^ ligand-interacting residues. Thus, these mutated sites do not directly influence ligand binding during reduction. Therefore, we next focused on the structural changes caused by the MDH mutants.

Molecular dynamics simulations of wild type MDH, F213V/F289L, and F213V/F289L/F356S were performed to examine changes to the secondary and tertiary structure. The secondary structure compositions among the three MDH variants were very similar. Alpha-helix compositions were 54, 52, and 53%, respectively, and beta-sheet compositions were all 10%. The three structures were superimposed to measure their backbone similarities, and the root mean square deviations (RMSDs) were 2.7 Å for the wild type-F213V/F289L and F213V/F289L-F213V/F289L/F356S pairs, and 2.5 Å for the wild type-F213V/F289L pair. Therefore, the three structures were very similar, and the mutations did not dramatically change the tertiary structure of MDH.

### Decameric structure of the MDH mutants

**Figure 4:**
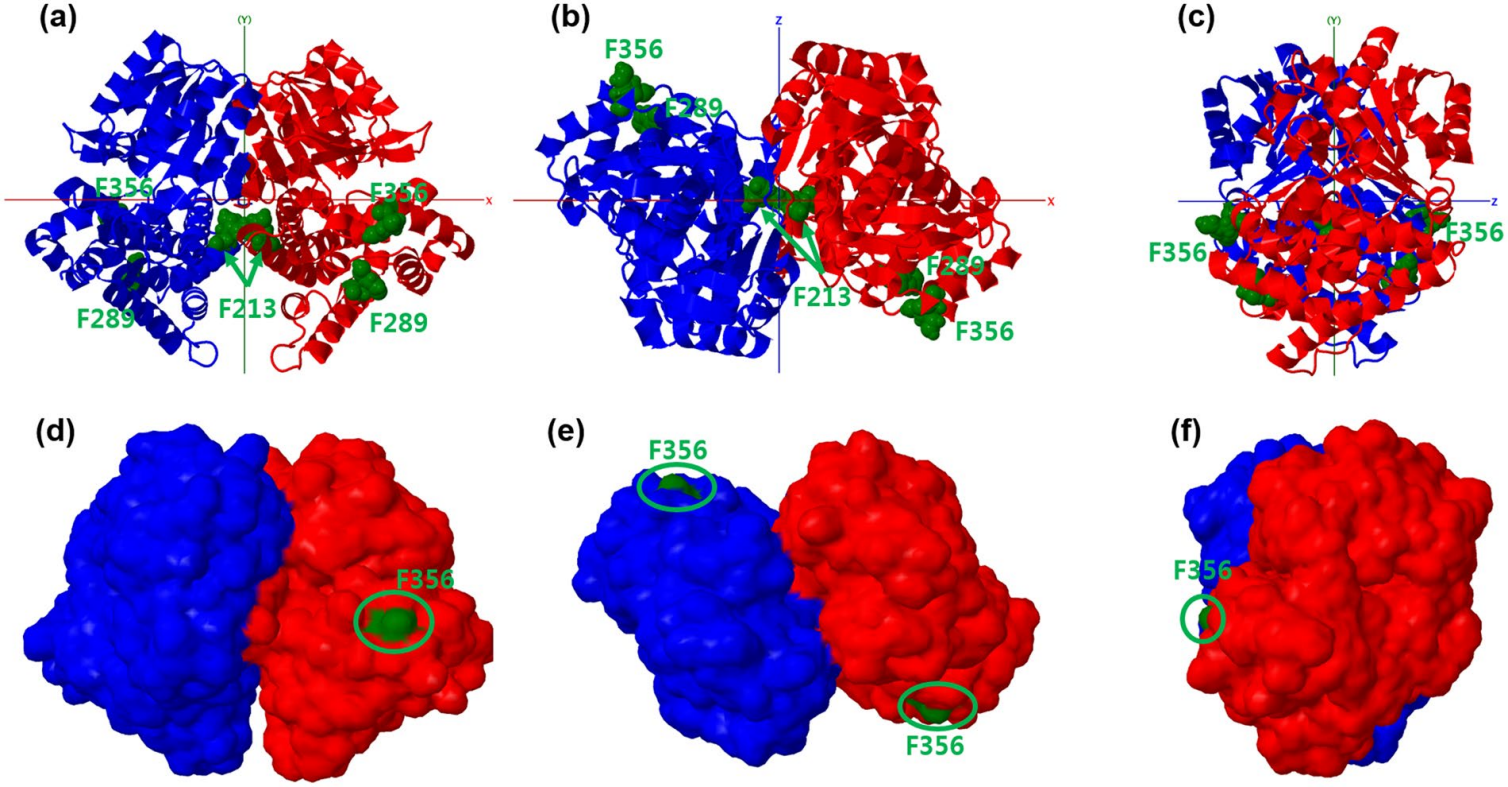
MDH dimer structure. Cartoon image (**a**–**c**) and molecular surface model (**d**–**f**) of the dimer. Chain A, red; chain B, blue. Mutation positions are shown in green space-fill models accompanied by the residue name and number. The dimer structures are oriented in the XY (**a**,**d**), XZ (**b**,**e**), and YZ (**c**,**f**) planes, respectively.

Because similar MDH proteins from thermotolerant *Bacillus* exist as decamers in electron microscopic images^24^, we prepared various oligomeric structures, including dimers, pentamers, and decamers. An oligomerization model was built from the monomer by PQR-SA, and was processed using the Chemistry at HARvard Macromolecular Mechanics (CHARMM) program^25^. Energy-minimized structures of various oligomers were analysed to determine how the mutant residues influenced oligomer stability and activity. Two decamer oligomerization pathways are possible: (1) two monomers form a dimer, and five dimers form a decamer (1 → 2 → 10 pathway), or (2) five monomers form a pentamer, and two pentamers form a decamer (1 → 5 → 10 pathway). Gel permeation chromatography suggested that most MDH structures existed as decamers and dimers (Fig. S4), suggesting the first pathway. Accordingly, first, a dimer structure was generated from the monomer homology structure. Two monomers were superimposed and structurally aligned based on known MDH dimer structure (PDB: 3OX4.A). Among mutants with only one amino acid change, only F213V showed higher catalytic activity than wild type MDH (Table 1). As shown in Fig. 4a–c, F213 was located in the interface of two monomers. Thus, the F213V substitution may increase reduction activity by influencing dimer formation. However, the other two mutations (F289 and F356) were not located on the dimer interface and therefore do not directly influence dimerization. F356 is located on the dimer surface, while F289 is buried in the structure (Fig. 4d–f). Pentamer and decamer structures were generated by five-fold symmetry rotation of five MDH monomers and dimers, by changing two geometrical variables, the inter-dimer distance of the centre of geometries of two neighbouring monomers, ranging from 40–50 Å in 2 Å increments, and the rotation angle of one of principal axes of the MDH monomer, ranging from 2–360° in 2° increments. The potential energy changes of the pentamer and decamer were calculated as functions of the inter-dimer distance and rotation angle to find the lowest energy conformation.

**Figure 5:**
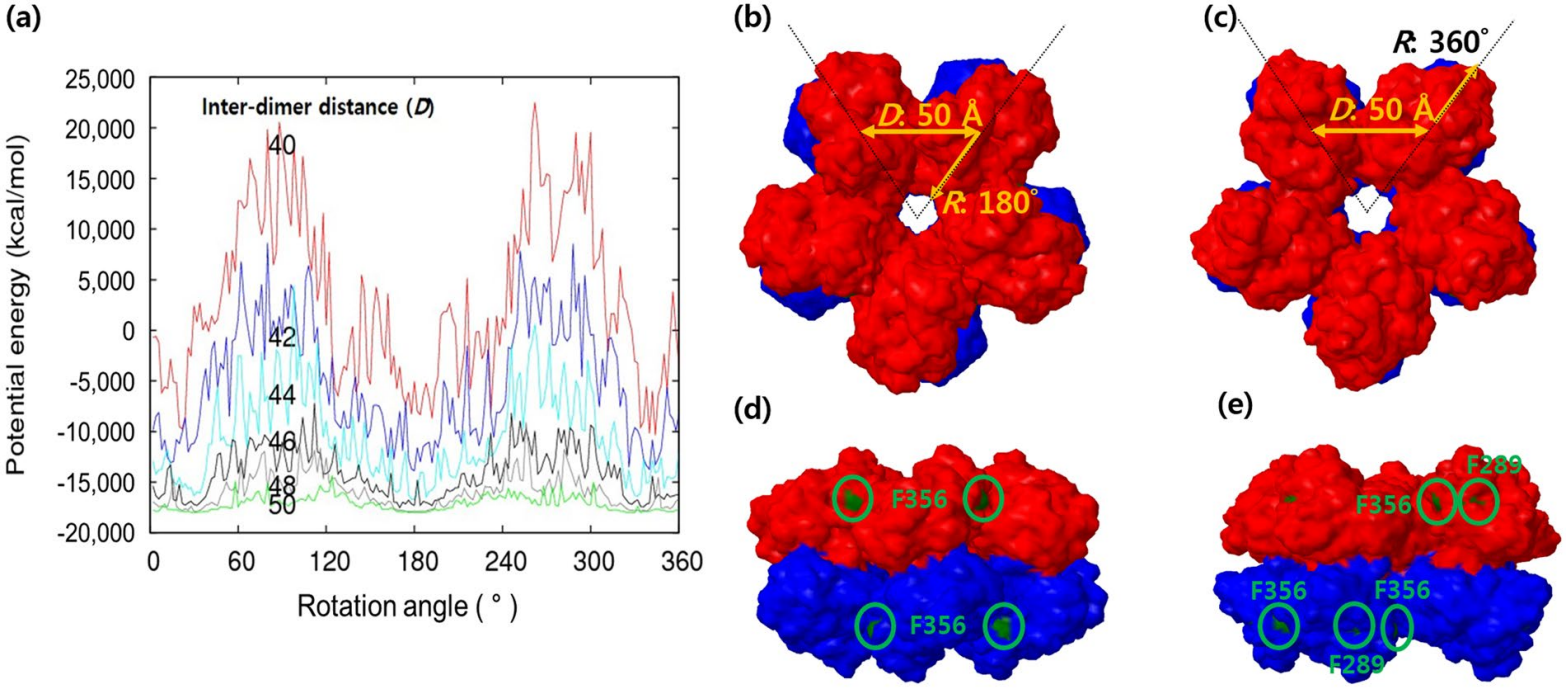
MDH decamer structure (**a**) Energy profile changes by rotation angle (*R:* x-axis) and inter-dimer distance (*D:* lines with different colours). (**b**) Top and side views of the decamer structure with (*R, D*) = (180°, 50 Å). (**c**) Top and side views of the decamer structure with (*R, D*) = (360°, 50 Å). The exposed mutation positions are shown in green and accompanied by residue numbers.

The generated model matched the electron microscopy data of Vonck *et al*.^24^ As shown in Fig. 5a, the measured energy profile indicated that two stable energy conformations existed, at rotation angles of 180° and 360° with 50 Å of inter-dimer distance. The decamer structures had unfavourable positive energy around 90° and 270° because neighbouring dimers collided when the inter-dimer distance was close, particularly at 40 Å. The side view of the decamer structure showed that the F289 and F356 residues were located in the proximity of the dimer-dimer interface. F289 was buried in the decamer model with the rotation angle of 180° and slightly exposed in the 360° rotation angle model. F289 and F356 were both located in the valley between dimers, and may influence decamer formation.

## Conclusions

We have successfully improved the formaldehyde reduction activity of MDH by directed evolution and screening, which might potentially be useful for the conversion of CO_2_ to methanol. We could suggest the positional influence of the mutant with computational modelling. The three substituted residues were located at MDH oligomerization interfaces. The substitutions may stabilise oligomerization, thereby increasing the formaldehyde reduction activity of MDH.

## Methods

### Strains, media, and materials

*E. coli* DH5α cells were used for general DNA recombinant techniques and BL21 (DE3) cells were used for MDH expression. DH5α and BL21 (DE3) competent cells were purchased from Real Biotech Corporation (Taipei, Taiwan). Cells were grown in 2YT medium (16 g yeast extract, 10 g tryptone, and 5 g NaCl per litre) supplemented with appropriate antibiotics. Restriction enzymes were purchased from New England Biolabs (Ipswich, MA, USA). DNA polymerase and ligase were from Takara (Shiga, Japan). Ni-affinity chromatography column was from GE Healthcare (Little Chalfont, UK). All chemicals except formaldehyde used in enzymatic reactions, including the cofactors NAD^+^ and NADH, were purchased from Sigma-Aldrich (St. Louis, MO, USA). And methanol-free 16% formaldehyde was from Thermo Scientific (Waltham, MA, USA).

### Directed evolution and mutant screening

The *E. coli* codon-optimized *mdh* (GenBank accession number M65004) from *B. methanolicus* C1^26^ with a C-terminal histidine tag was synthesized by Bioneer (Daejeon, Korea) and randomly mutated using a Diversify® PCR Random Mutagenesis Kit (Clontech, Mountain View, CA, USA) with the primers mdh-forward (5′-GACATATGACAAACTTTTTCATTCC-3′) and mdh-reverse (5′-AACTCGAGCAGAGCGTTTTTGATG-3′) to produce 2–8 bp substitutions per kb, according to the manufacturer’s instructions. Underlines indicate restriction enzyme sites: CATATG (NdeI) and CTCGAG (XhoI). The wild type and mutated genes were digested with NdeI/XhoI, and ligated into pET-21b (Novagen, Billerica, MA, USA) digested with the same enzymes. *E. coli* DH5α was transformed with ligation mix and spread on 2YT agar plate supplemented with ampicillin. The size and quality of the constructed library were analysed as described previously^27^. BL21 (DE3) cells were transformed with the vectors, which were prepared from DH5α, by electroporation and cultivated on agar plate or in 2YT broth containing 100 μg/mL ampicillin at 37 °C. The number of transformants was calculated from the number of colonies on plates. To express MDH, 0.1 mM of isopropyl β-D-1-thiogalactopyranoside (IPTG) was added when the optical density at 600 nm (OD_600_) reached 0.6. After MDH expression for 1 h, 3 mM formaldehyde was added. After an additional 24 h incubation, the medium was streaked on 2YT agar plates containing 100 μg/mL ampicillin. Plasmids were isolated from single colonies and sequenced.

To analyse the effects of the mutant MDHs on formaldehyde reduction, the growth of cells harbouring wild type and mutant MDH variants was monitored in media containing formaldehyde. After induction of MDH expression for 1 h, 3 mM formaldehyde was added, and the OD_600_ was measured every 2 h, until formaldehyde was detoxified to levels enabling the resumption of cell growth. To exclude additional mutations within the gene, the mutant *mdh* genes were sequenced at every step.

### Purification of MDH variants

BL21 (DE3) cells harbouring plasmids encoding wild type or mutant MDH were cultivated in 2 L 2YT broth at 37 °C and 180 rpm. When the OD_600_ reached 0.4, 0.1 mM IPTG was added. After 24 h, cells were harvested and resuspended in Buffer A (20 mM Tris pH 7.5, 0.5 M NaCl). Resuspended cells were disrupted by sonication (SONIFIER250, Branson, CT, USA). Then, the crude solution was centrifuged at 10,000 × *g* for 30 min. The supernatant was filtered through a 0.45 μm Minisart® filter (Sartorius Stedim Biotech, Gӧettingen, Germany). The filtered fraction was applied to a HisTrap™ FF Nickel affinity column (GE Healthcare) equilibrated with Buffer A. The bound proteins were eluted with 250 mM imidazole (pH 7.5). To eliminate remaining imidazole in the eluted protein solution and replace the buffer for subsequent experiments, an Amicon® Ultra-15 Centifugal Filter (Millipore, Bedford, MA, USA) was used. After purification, the apparent molecular masses of MDH were determined on a 4–12% gradient sodium dodecyl sulphate-polyacrylamide gel (NOVEX, San Diego, CA, USA). Protein concentrations were determined using the BCA™ Protein Assay Kit (Pierce, Rockford, IL, USA) using bovine serum albumin as a standard.

### Gel permeation chromatography of native MDH

Purified MDHs were diluted in 100 mM potassium hydrogen phosphate buffer (PHPB; pH 7.5) and the final concentration was adjusted to 1 mg/mL for injection into a Superose 12 10/300 GL column (GE healthcare) on an AKTA fast protein liquid chromatography system at 4 °C. The column was equilibrated with PHPB. Purified MDH (0.5 mL) was loaded into the column and eluted at a flow rate of 1 mL/min. A calibration curve was prepared with a protein standard marker (#151–1901) purchased from Bio-Rad (Hercules, CA, USA).

### Formaldehyde reduction assay

Formaldehyde reduction activity of MDH was assayed spectrophotometrically by detecting decreased NADH, as described previously^17,24,28^. Reactions were started by adding 100 μg of MDH to a reaction solution (500 μL) containing 100 mM PHPB, 0.6 mM NADH, and 3 mM formaldehyde, and the decrease in NADH was measured at 340 nm at room temperature.

The kinetic parameters of MDH with respect to formaldehyde reduction were determined in 100 mM PHPB (500 μL) containing 0.3 mM NADH for 20 min at 50 °C. The MDH content was fixed at 10 μg, while the concentration of formaldehyde varied from 0.1–10 mM. The initial rate of the reactions was determined by measuring the change in NADH. Experiments were performed in triplicate and repeated three times.

### HPLC measurements of methanol and formaldehyde

The methanol produced from formaldehyde by MDH (100 μg) in a reaction solution (500 μL) containing 100 mM PHPB, 50 mM NADH, and 30 mM formaldehyde for 1 h at room temperature was measured using an Agilent HPLC station (Santa Clara, CA, USA), equipped with an Aminex HPX-87H column (300 × 7.8 mm, Bio-Rad) and a refractive index detector. HPLC-grade 0.01 N H_2_SO_4_ was used in the mobile phase, at a flow rate of 0.6 mL/min at 60 °C. A standard curve was generated with formaldehyde and methanol.

### 3D structure modelling

A protein structure for MDH from *B. methanolicus* C1 was built using homology modelling. HHblits^29^ was used to find suitable template structures in the PDB. The PQR-SA protocol^30^ was used to generate the homology structure from identified template structures by minimizing the target energy potential, consisting of the stereochemical energy, derived multiple alignment restraint energy from the PQR-SA protocol, and statistical torsion angle potential^31^. We predicted binding sites in the generated structure using the DPSP method (http://psb.kobic.re.kr/dpsp/).

To examine the influence of mutant residues on the structure, 10 ns molecular dynamics simulations of wild type and mutant MDHs were performed using CHARMM^25^. To consider the solvation effect, a generalized Born model with a simple switch function was used^32^. The initial structure, which was the lowest energy structure generated in homology modelling, was energy-minimized, and we performed Langevin dynamics with 2 fs intervals at room temperature. The RMSDs, secondary structure measurements, and time profiles of surface area changes were measured.

## Electronic supplementary material

Supplementary Information
