# Supplementary material for: Development of Bacillus methanolicus methanol dehydrogenase with improved formaldehyde reduction activity
Source: Sci Rep. 2018 Aug 20;8:12483. doi: 10.1038/s41598-018-31001-8 (PMC6102214; doi:10.1038/s41598-018-31001-8)

Development of *Bacillus methanolicus* methanol dehydrogenase with improved formaldehyde reduction activity

Authors

Jiyeun Yi[1,2,¶], Jinhyuk Lee[3,4,¶], Bong Hyun Sung[2,4,¶], Du-Kyeong Kang[2,4], GyuTae Lim[3,4], Jung-Hoon Bae[2], Seung-Goo Lee[4,5], Sun Chang Kim[1,*], and Jung-Hoon Sohn[2,4,*]

Affiliations

[1]*Department of Biological Sciences, Korea Advanced Institute of Science and Technology, Daejeon 34141, South Korea*

[2]*Cell Factory Research Center, Korea Research Institute of Bioscience and Biotechnology, Daejeon 34141, South Korea*

[3]*Genome Editing Research Center, Korea Research Institute of Bioscience and Biotechnology, Daejeon 34141, South Korea*

[4]*School of Biotechnology, Korea University of Science and Technology, Daejeon 34113, South Korea*

[5]*Synthetic Biology and Bioengineering Research Center, Korea Research Institute of Bioscience and Biotechnology, Daejeon 34141, South Korea*

Supplementary Information

Figure S1. Time course analysis of methanol production from formaldehyde by MDHs

Figure S2. Generated homology model of wild type MDH

Figure S3. MDH binding site prediction

Figure S4. Gel permeation chromatography of wild type MDH

Figure S1. Time course analysis of methanol production from formaldehyde by MDHs

The formaldehyde (30 mM) was converted to methanol by wild-type MDH (WT) and MDH variant F213V/F289L/F356S for 4 hours, respectively. The concentration of formaldehyde **(a)** and methanol **(b)** was measured every hour. The data represent the mean value and standard deviation of three independent experiments.

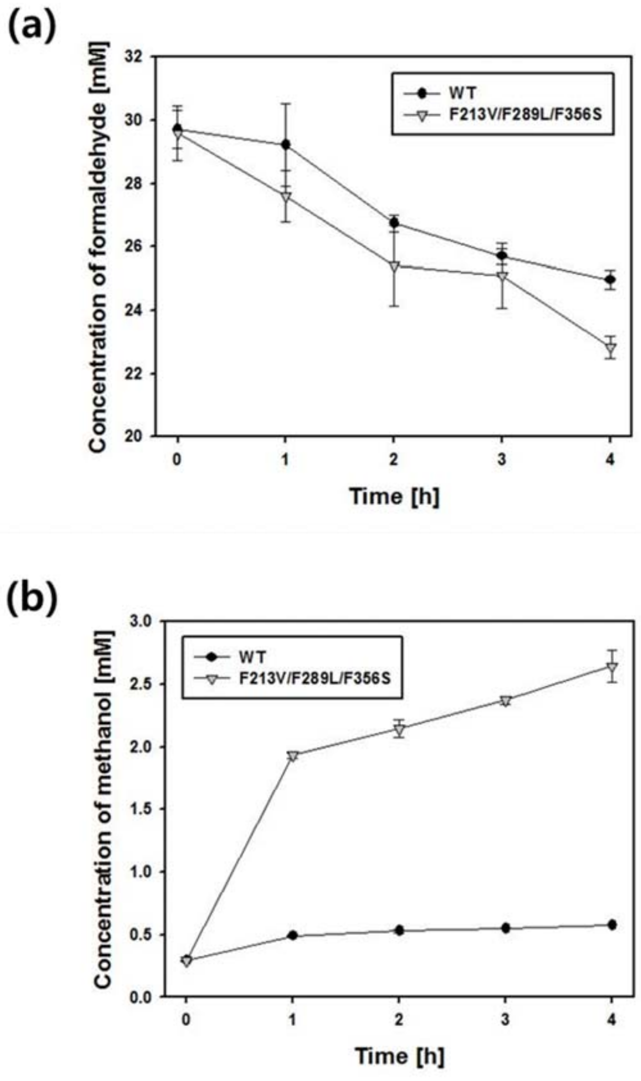

Figure S2. Generated homology model of wild type MDH

(a) A cartoon structure shaded by secondary structure schemes (alpha helix, red; beta sheet, yellow; random coil, blue). The mutation positions, F213, F289, and F356, are shown as green space-filled models. **(b)** The radius of gyration of MDH is shown compared to that of high resolution X-ray structures and a fitting curve. Rgyr, radius of gyration value. **(c)** The protein quality scoring scheme of the homology structure. The eleven scores were normalized from 0 (best, inbound) to 1 (worst, outbound), as calculated by various programs, including Molprobity, Procheck, and Whatcheck. The inner grey circle represents the normalized score region within 50% of high-resolution X-ray structures. Protein structure was shown using the ribbon structure of the Jmol program, and graphs were plotted using GNU plot.

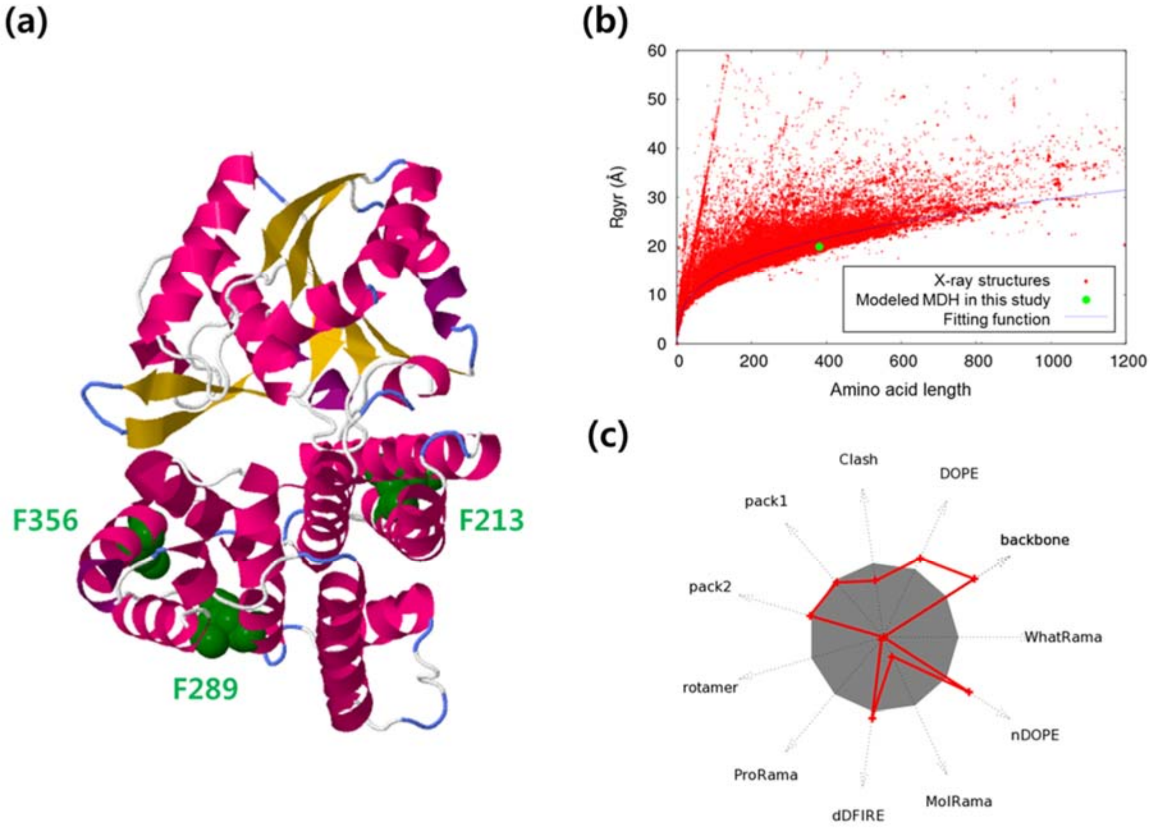

Figure S3. MDH binding site prediction

Predicted NAD^+ binding sites are shown as yellow spheres, and the three mutations are shown as green sphere models with the residue names and numbers (left). Heat-map obtained from Dockable Pocket Site Prediction calculations (right). The x- and y-axes represent the identified pockets (with arbitrary numbers) and ligands (named as in the protein data bank), respectively. Darker red indicates a higher possibility of ligand binding.

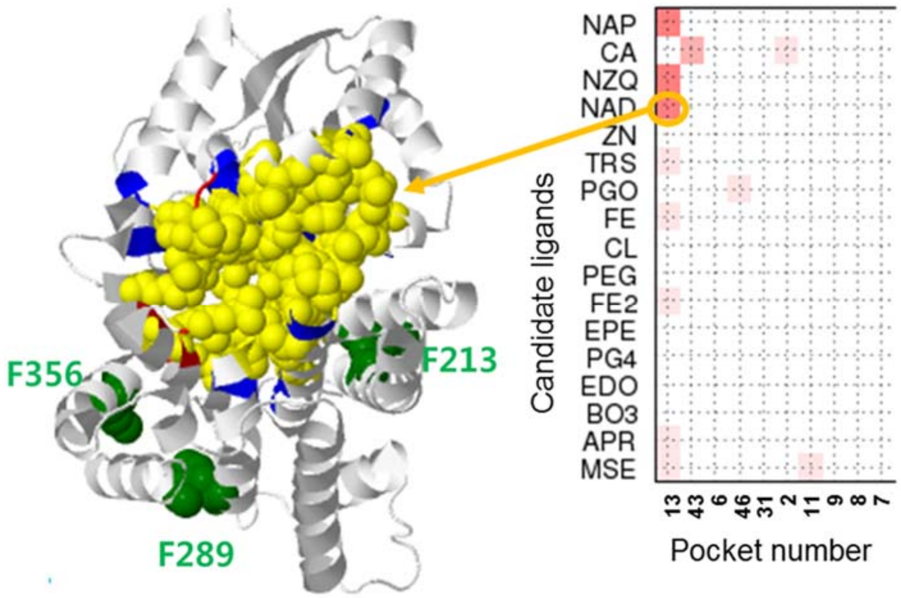

Figure S4. Gel permeation chromatography of wild type MDH

(a) Three main peaks of native MDH were compared with standard proteins. The y-axis represents the molecular weight of the protein, expressed as a log scale. The x-axis indicates the elution volume from gel filtration chromatography. **(b)** The y-axis is the UV curve of native MDH, and the x-axis represents the volume eluted from the column.

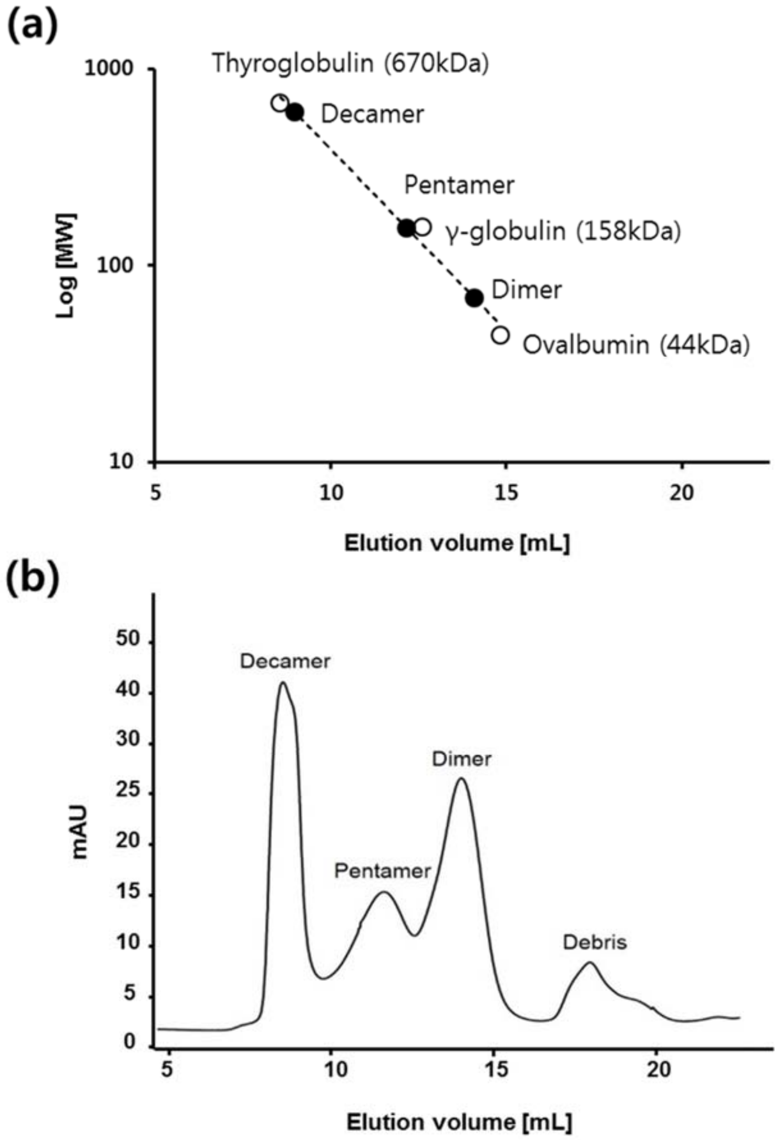

Supplement: Supplementary file 1 — Supplementary Information [file 41598_2018_31001_MOESM1_ESM.pdf]
